# Fiber-Enhanced Stimulated Raman Scattering and Sensitive Detection of Dilute Solutions

**DOI:** 10.3390/bios12040243

**Published:** 2022-04-14

**Authors:** Li Guo, Jing Huang, Yaxin Chen, Bohan Zhang, Minbiao Ji

**Affiliations:** 1 State Key Laboratory of Surface Physics and Department of Physics, Human Phenome Institute, Academy for Engineering and Technology, Key Laboratory of Micro and Nano Photonic Structures (Ministry of Education), Fudan University, Shanghai 200433, China; 19110190067@fudan.edu.cn (L.G.); jing_huang@fudan.edu.cn (J.H.); yxchen19@fudan.edu.cn (Y.C.); 20110190081@fudan.edu.cn (B.Z.); 2 Yiwu Research Institute, Fudan University, Chengbei Road, Yiwu 322000, China

**Keywords:** stimulated Raman scattering, fiber enhanced, spectroscopy, biosensing

## Abstract

Stimulated Raman scattering (SRS) is known to gain coherent amplification of molecular vibrations that allow for rapid and label-free chemical imaging in the microscopy setting. However, the tightly focused laser spot has limited the detection sensitivity, partly due to the tiny interaction volume. Here, we report the use of metal-lined hollow-core fiber (MLHCF) to improve the sensitivity of SRS in sensing dilute solutions by extending the light–matter interaction volume through the fiber waveguide. With a focusing lens (100 mm FL) and 320 μm diameter fiber, we demonstrated an optimum enhancement factor of ~20 at a fiber length of 8.3 cm. More importantly, the MLHCF exhibited a significantly suppressed cross-phase modulation (XPM) background, enabling the detection of ~0.7 mM DMSO in water. Furthermore, the relationship between fiber length and SRS signal could be well explained theoretically. The fiber-enhanced SRS (FE-SRS) method may be further optimized and bears potential in the sensitive detection of molecules in the solution and gas phases.

## 1. Introduction

Raman scattering spectroscopy, a fundamental technique based on molecular rotation and bond vibration, is used to provide intrinsic molecular information of samples noninvasively [1]. Spontaneous Raman scattering is known to be limited by the weak scattering cross-section that hinders its application for sensitive detection and biomedical imaging. A few types of techniques have been developed to boost the scattering efficiency including surface-enhanced Raman scattering (SERS) via plasmonic enhanced local electric field [2], UV-enhanced Raman (UV-Raman) via electronic resonance [3], and coherent Raman scattering (CRS) via coherent nonlinear optical amplification [4,5,6].

Stimulated Raman scattering (SRS) is a type of CRS with well-preserved spectral line shapes, in contrast to the other—coherent anti-Stokes Raman scattering (CARS) with distorted spectra [7,8,9]. In addition, SRS signal is linearly proportional to molecular concentration, whereas CARS is nonlinear; hence, SRS is preferred for quantitative chemical analysis [9,10,11,12]. Most commonly adapted in the microscopy mode, SRS is attracting growing interest for rapid chemical imaging in various research areas including label-free tissue histology, metabolic imaging, drug delivery, and material science [6,13,14,15,16,17,18,19,20,21,22,23]. However, SRS microscopy has limited detectability for low-concentration analytes, with a typical detection limit of a few mM for small molecules such as DMSO [24]. Given the signal of *I_SRS_* ∝ *NI_p_I_S_*, although the tightly focused laser beams gain much in power densities of the pump *I_p_* and Stokes *I_S_* beams, it sacrifices the number of probed molecules *N* because of the small focal volume. Since the excitation laser powers cannot exceed the damage threshold, the detection of dilute solutions remains a challenge. Furthermore, as the analyte concentration becomes lower, the non-Raman background (e.g., cross-phase modulation—XPM) begins to overwhelm the true SRS signal, causing signal-to-background issues [8,25,26].

An alternative way to gain the signal is to increase the number of interacting molecules. For spontaneous Raman, fiber-enhanced Raman spectroscopy (FERS) has been developed for the real-time detection of molecules filled in hollow-core optical fibers [27]. The fiber core waveguide confines the optical fields and effectively extends the interaction volume with molecules. Hollow-core photonic crystal fiber (HC-PCF) [28] and metal-lined hollow-core fiber (MLHCF) [29,30,31] are the two major types of fiber used for detection, which have their advantages and disadvantages [32]. For HC-PCF, the low transmission loss and large numerical aperture (NA) allow for the efficient generation of spontaneous and stimulated Raman scattering [33,34,35], and it has demonstrated potential in the sensitive sensing of biomolecules and pharmaceuticals [36,37,38,39]. However, the small core diameter of HC-PCF tends to generate detrimental nonlinear optical signals such as the XPM and four-wave mixing (FWM). Moreover, it also restricts the efficient transport of liquid or gas, limiting the potential applications for fast sensing. Furthermore, liquid-filled HC-PCF results in a much narrowed optical bandwidth and increased transmission loss [37,40], introducing additional difficulties for SRS detection. In contrast, MLHCF with a larger core diameter enables rapid chemical exchange under normal pressure [29,41], and the metal surface reflection ensures a broad bandwidth; hence, MLHCF may be more suited for real-time sensing of gas or liquid with SRS. Despite extensive studies on hollow-core fiber sensing with spontaneous Raman scattering and fluorescence measurements [38,42], the properties of the SRS signal of molecules in hollow-core fibers remain unexplored.

In this work, we demonstrated the use of MLHCF to enhance SRS signal for detecting low-concentration solutions. Our results showed the competing effects of SRS signal amplification and power loss with increasing fiber length, which agreed well with the theoretical model. In our particular system, composed of a 100 mm focal length lens and a 320 μm diameter MLHCF with a sample volume of 6.7 μL, a maximum enhancement factor of ~20 was achieved with a fiber length of 8.3 cm. More interestingly, we found the XPM background was significantly suppressed in MLHCF. A detection limit down to ~0.7 mM of DMSO/water solution was achieved in the loose focusing geometry.

## 2. Materials and Methods

### 2.1. Spontaneous Raman Spectroscopy

Spontaneous Raman spectra from samples were collected by a home-built Raman spectrometer. In the setup, a helium–neon (HeNe) laser at 633 nm was used to excite the sample under the microscope via a 40X objective, and the emitted Raman signal was sent to a monochromator (iHR320, Horiba) and collected by a charge-coupled device (CCD) camera (Symphony, Horiba).

### 2.2. SRS Spectroscopy

**Figure 1:**
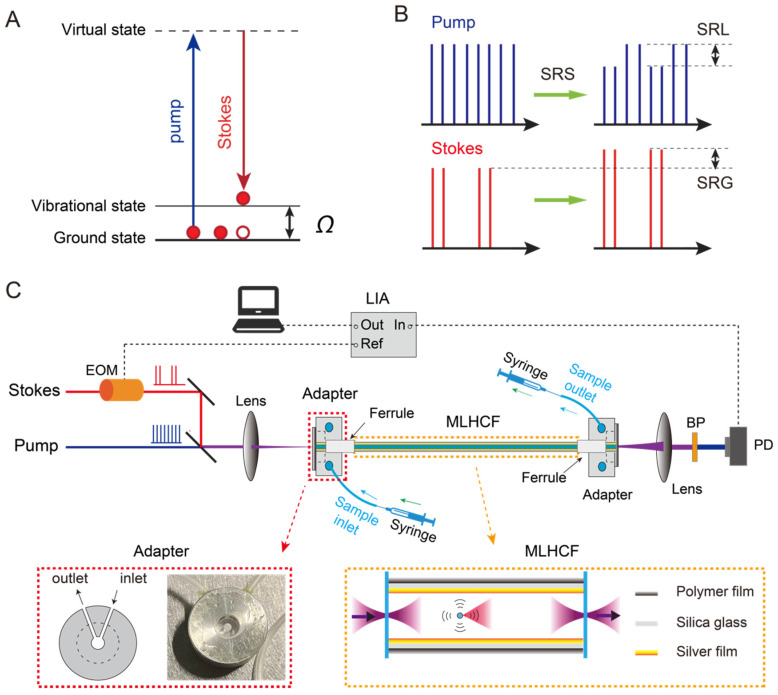
Experimental design: (**A**) energy diagram of stimulated Raman scattering (SRS); (**B**) illustration of the laser intensity changes as a result of stimulated Raman loss (SRL) and stimulated Raman gain (SRG); (**C**) optical layout of the fiber-enhanced stimulated Raman scattering setup. EOM: electro-optic modulator; MLHCF: metal-lined hollow-core fiber; BP: bandpass filter; PD: photodiode; LIA: lock-in amplifier.

As a nonlinear optical process, SRS requires two synchronized pulsed lasers, the pump ($\omega_{p}$) and Stokes ($\omega_{s}$) beams. When the frequency difference between two beams matches the bond vibration (Ω) of interest (i.e., $\omega_{p}-\omega_{s}=\Omega$), the molecules inside the laser focal volume are coherently excited (Figure 1A), with orders of magnitude (~10^3^–10^5^) gain of efficiency compared with spontaneous Raman scattering. In our study, a commercial femtosecond optical parametric oscillator (OPO, Insight DS+, Newport, CA, USA) with dual outputs was used as the laser source. The fundamental 1040 nm beam was used as the Stokes beam (~150 fs), and the tunable OPO output (680–1300 nm, 120 fs) was used as the pump beam. To obtain a higher spectral resolution with the “spectral focusing” mode, high-dispersive glass rods (SF57) were used to chirp the pump and Stokes beams to ~2.3 and ~1.2 ps, respectively [43]. The Stokes beam was modulated by an electro-optical modulator (EOM, EO-AM-R-20-C2, Thorlabs) at 20 MHz and aligned with pump beam through a dichroic mirror (DMSP1000, Thorlabs) and focused into the hollow-core fiber by a lens (AC254-100-B, Thorlabs), while the output was collimated by another lens, through a bandpass filter (CARS ET890/220, Chroma) to pass the pump beam, and sent to a homemade back-biased photodiode (PD). The stimulated Raman loss (SRL, Figure 1B) signal was collected by a lock-in amplifier (LIA) (HF2LI, Zurich Instruments) to demodulate the 20 MHz signal from the PD (Figure 1C). The power of the pump and Stokes beams incident into the focusing lens were kept at 200 and 100 mW, respectively. The SRS spectra were measured by scanning the time delay between the pump and Stokes pulses, with 50 ms integration time for each spectral point.

### 2.3. Fiber Adapter

As shown in Figure 1C, our designed fiber adapter was made of aluminum with a small chamber as the liquid cell, an inlet and an outlet side-holes to inject or extract the sample. The front surface was sealed with a coverslip, and the back surface was connected with a ceramic ferrule to guide the MLHCF into the liquid cell. The adapters were installed at both ends of the fiber to stably mount the MLHCF. Precise micrometer alignment of the optical fiber relative to the focused laser beam was achieved by mounting the adapter onto a 3-axis microblock stage (MBT616D, Thorlabs). The syringes were used to load or unload the liquids by pushing or pulling.

### 2.4. MLHCF

The metal-lined hollow-core fiber (MLHCF, Do-Ko engineering VSS320450) had an inner diameter of 320 μm and an outer diameter of 450 μm. The inner coating of the MLHCF was silver film, while the outer cladding was polymer film. In our study, the fiber was cut into lengths of 2.7, 3.2, 4.1, 8.3, 12.1, 16.5, 22.9, 29.7, and 49.5 cm.

### 2.5. Chemicals

Ethanol was purchased from Titan Scientific Co., Ltd. (Shanghai, China). DMSO was purchased from Yuanye Bio-Technology Co., Ltd. (Shanghai, China).

## 3. Results and Discussion

### 3.1. Light Coupling and Transmission Loss in MLHCF

The pump and Stokes beams could be treated as Gauss beams with ~2 mm diameter, and after focusing with a 100 mm FL lens, the focal spot size was ~32 μm in diameter, which was much smaller than the inner diameter of the MLHCF (320 μm). In addition, the numerical aperture (NA) of the lens (~0.01) was smaller than the effective NA of the MLHCF (0.05), and the coupling efficiency of the beams into the fiber could reach to approximately 100%. When the core of the MLHCF was filled with liquid sample, the absorption of near infrared (NIR) light by the long-path liquid resulted in power loss.

To quantify the attenuation coefficient (*α*), we measured the laser power losses caused by the fiber. The input power (*P*_0_) and transmitted power (*P_T_*) of the pump and Stokes beams before and after the fibers with different lengths (*L*) were measured, and the transmission efficiency *η* = *P_T_*/*P*_0_ of the fibers filled with sample solutions could be calculated. As shown in Figure S1, air-filled hollow-core fiber showed minimal attenuation. In contrast, the attenuation coefficients of the laser beams through the ethanol-filled fiber were found to be $\alpha_{p}=8.1\;m^{-1}$ and $\alpha_{s}=13.7\;m^{-1}$ at the wavelengths of 952 and 1040 nm, respectively, which qualitatively agree with the absorption spectrum of ethanol [44].

### 3.2. Fiber Length-Dependent SRS Signal

**Figure 2:**
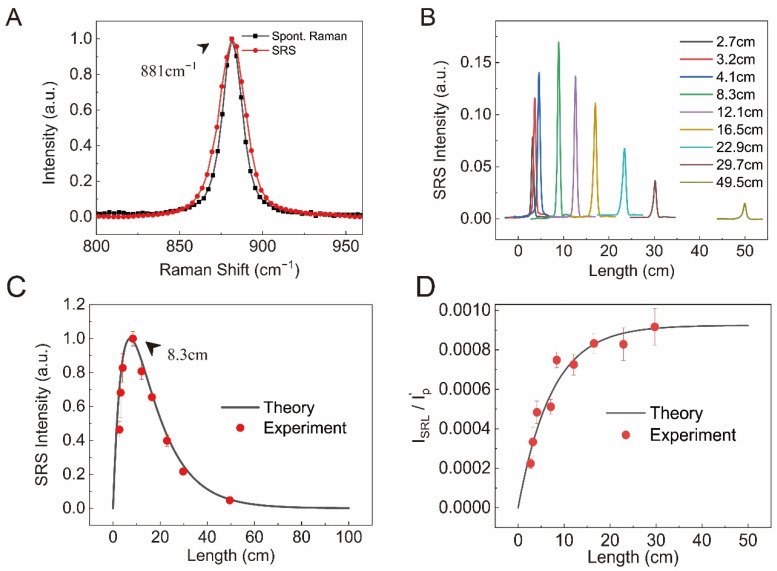
(**A**) Spontaneous Raman and SRS spectra of ethanol at ~881 cm^−1^; (**B**) SRS spectra of ethanol with different fiber lengths; (**C**) the normalized experimental data versus the theoretical prediction of the relationship between the SRS peak intensity and the fiber length; (**D**) the experimental data and the theoretical prediction for the fractional change of SRS to the fiber length.

The principle and optical design of the fiber coupled SRS is illustrated in Figure 1. We first characterized the dependence of SRS on fiber length with ethanol (95 *v*/*v*%, 16.3 M), targeting at the Raman band of ~881 cm^−1^ (C-C-O symmetric stretching vibration modes) [45]. The SRS spectrum of ethanol was found to agree well with its spontaneous Raman spectrum (Figure 2A and Figure S2). In our study, the additional dispersion of liquids did not seem to significantly affect the SRS spectra. Figure 2B shows the measured SRS spectra of ethanol with different fiber lengths, ranging from 2.7 to 49.5 cm. SRS intensity demonstrated an initial increase with increasing fiber length, followed by a decay as the fiber further lengthened (Figure 2C). Such a behavior indicates a competing effect between a gain and a loss mechanism as the light waves propagate through the liquid.

It is known that the SRS process results in the annihilation of the pump photons and the generation of Stokes photons, while the net photon energy loss is converted to the molecular vibrational excited states (Figure 1A). As a result, the pump beam experiences a reduction in intensity—stimulated Raman loss (SRL), and the Stokes beam experiences an increase in intensity—stimulated Raman gain (SRG) [5]. Further taking the laser attenuation into account, the coupled equations of the pump and Stokes beams intensities can be written as [46]:

(1) $$\frac{dI_{p}}{\mathrm{dz}}=-\frac{\omega_{p}}{\omega_{s}}g_{R}I_{p}I_{s}-\alpha_{p}I_{p}$$

(2) $$\frac{dI_{s}}{\mathrm{dz}}{=g}_{R}I_{p}I_{s}-\alpha_{s}I_{s}$$

where $g_{R}$ is the Raman-gain coefficient, and *α* is the attenuation constant of the fiber. Equation (1) represents the SRL and absorption processes of the pump beam, and Equation (2) represents the SRG and absorption processes of the Stokes beam.

Under the approximation of *α_p_* = *α_s_* = *α*, the equations could be solved analytically (Supplementary Materials Note S1), and the SRL signal is the differential intensity of the pump with and without Stokes beam; hence, it can be calculated as:

(3) $$I_{SRL}=I_{p}(0)e^{-\alpha z}-I_{p}(0)(1+\rho \frac{I_{s}(0)}{I_{p}(0)})e^{-\alpha z}/G(z)$$

where:

(4) $$G(z)=1+\rho \frac{I_{s}(0)}{I_{p}(0)}e^{-F(z)}$$

(5) $$\mathrm{and}\;F(z)=\rho g_{R}(\frac{I_{p}(0)}{\rho }+I_{s}(0))\left(1-e^{-\alpha z}\right)/\alpha$$

Here, $I_{s}(0)$ and $I_{p}(0)$ are the initial intensities of the beams at z=0. Moreover, the fractional change in the SRL relative to the transmitted pump beam intensity in the absence of the Stokes (*I_p_′*) could be calculated as:

(6) $$I_{SRL}/I_{p}{}^{\prime }=1-(1+\rho \frac{I_{s}(0)}{I_{p}(0)})/G(z)$$

These analytical solutions provide the approximated results of the SRS signal dependence on fiber length. In our true experimental measurements, *α_p_* = *α_s_* was not satisfied, and Equations (1) and (2) needed to be solved numerically. By fitting the SRS intensity data with the numerical solution, the good agreement between the theoretical and experimental results can be seen (Figure 2C). The fractional change in SRS could also be analyzed in Figure 2D, indicating that although the absolute SRS intensity decays almost exponentially as the fiber length becomes much longer, and the fractional change reaches a maximum constant, in our case, ~10^−3^. By fitting the fractional change in SRS, the Raman gain coefficient of the particular mode can be extracted as $g_{R}=0.058\;m^{-1}\cdot W^{-1}$. The slight difference between the numerical and analytical solutions can be found in Figure S3. Our experimental results indicated that. the maximum SRS intensity occurred with a fiber length of 8.3 cm, close to the theoretical value of ~7.0 cm (Figure S3).

### 3.3. SRS Enhancement of Ethanol Detection in the Fingerprint Region

**Figure 3:**
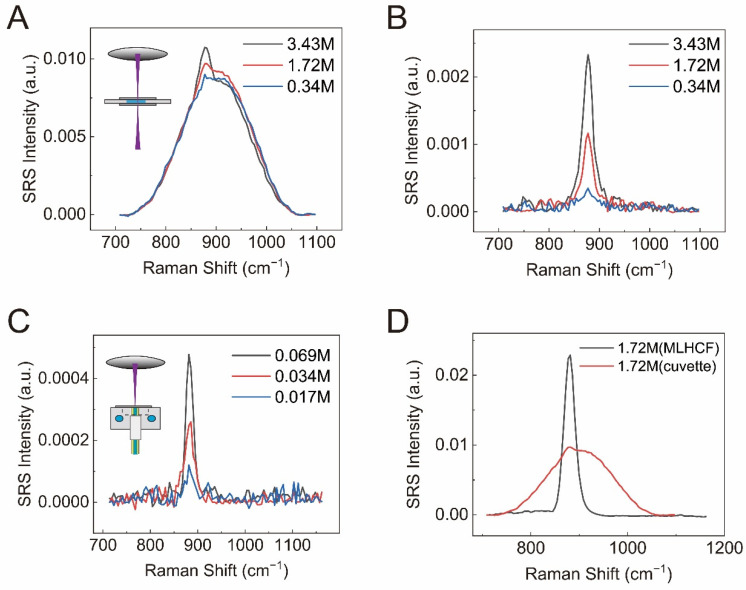
(**A**) Raw SRS spectra of ethanol with different concentrations for measurements in the cuvette; (**B**) background-corrected SRS spectra of ethanol with different concentrations for measurements in the cuvette; (**C**) background-removed SRS spectra (881 cm^−1^) of ethanol with different concentrations via 8.3 cm long MLHCF; (**D**) SRS spectra of 1.72 M ethanol from the cuvette and the 8.3 cm fiber.

To quantify the enhancement factor, we needed to first define it. Here we took the SRS intensity measured with the same focusing lens of the same sample in a 1 mm thick cuvette as the reference. The enhancement factor was calculated as the ratio between the SRS intensity with the MLHCF and the intensity with the cuvette. The SRS spectra of ethanol at ~881 cm^−1^ were measured to represent the case in the fingerprint region, which is known to be relatively weak compared with the high-frequency C-H stretching region. The experimental results are shown in Figure 3.

For the measurements in the cuvette, the SRS showed poor detectability for the fingerprint Raman mode, and the weak signal was quickly overtaken by the XPM background at reduced concentrations (Figure 3A). Even by removing the background, the detectable concentration was limited to as large as ~340 mM (Figure 3B). The XPM background is the nonlinear optical effect that alters the refractive index of the medium by intense light pulses, changing its spectral property as well as the spatial profile. It can be concluded that the XPM is a significant issue for detecting dilute solutions, in both the microscopy mode and in the single-focusing lens mode.

In strong contrast, the detectability of SRS with MLHCF was greatly enhanced. SRS signal through a MLHCF (8.3 cm long) was enhanced almost 20 times compared with the result from the 1 mm thick cuvette. As shown in Figure 3C, the detection limit could reach down to ~17 mM. The linear dependence of signal intensity on ethanol concentration was shown in Figure S4. In addition to the signal enhancement, MLHCF exhibited much suppressed XPM background, which is of great importance for SRS detection. The suppression of the nonlinear XPM might be because of the much weaker power density in the large-core fiber waveguide. As illustrated in Figure 3D, for 1.72 M ethanol, while the SRS spectrum from the cuvette was already overwhelmed by the large background, the spectrum from the fiber had a negligible XPM background and showed a single narrow Raman band at approximately 881 cm^−1^.

### 3.4. SRS Enhancement of DMSO in the High-Frequency Region

**Figure 4:**
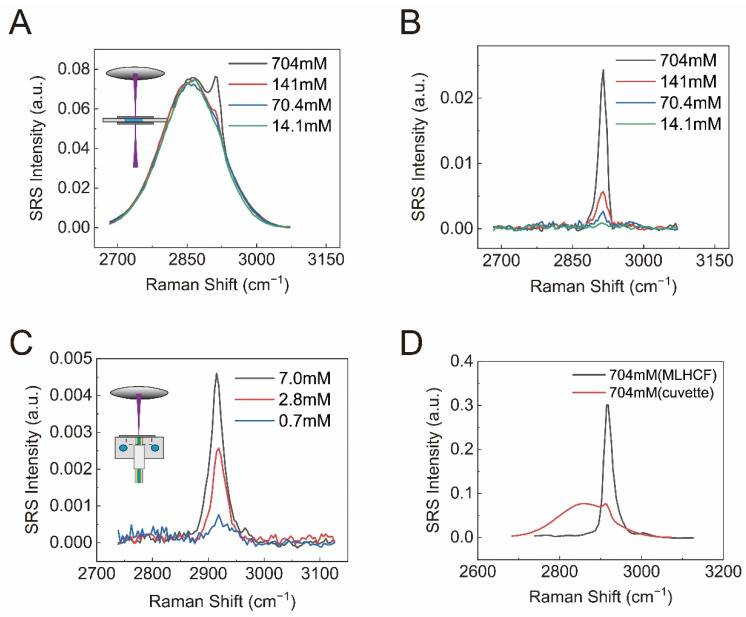
(**A**) Original SRS spectra of DMSO/water solutions in the cuvette with different concentrations; (**B**) background-corrected SRS spectra of DMSO/water solutions in the cuvette with different concentrations; (**C**) background-corrected SRS spectra (2915 cm^−1^) of DMSO with different concentrations via MLHCF with a length of 8.3 cm; (**D**) SRS spectra of 704 mM DMSO from the cuvette and the 8.3 cm fiber.

To further test SRS spectra in the high-frequency region using MLHCF, we took measurements of dimethyl sulfoxide (DMSO) dissolved in water. The characteristic peak at ~2915 cm^−1^ (C-H band, antisymmetric stretching) was chosen to quantify SRS intensity of DMSO (Figure S2). The raw SRS spectra of the DMSO/water solutions sealed in the cuvette with varying concentrations are presented in Figure 4A. Although the overall SRS signal in this spectral range was approximately an order of magnitude larger than the intensity in the fingerprint region, obvious XPM background still becomes dominant for low-concentration solutions. After background correction, the detection limit was estimated to be ~14.1 mM as demonstrated in Figure 4B. As for MLHCF (8.3 cm long), the detectable concentration could be pushed down to ~0.7 mM (Figure 4C). The linear concentration dependence of SRS intensity is plotted in Figure S4, indicating the capability of quantitative measurements. Similar to the fingerprint region results, the suppression of the XPM background was also significant (Figure 4D). The combined advantages of remarkable signal enhancement and efficient background reduction ensured that fibre-enhanced SRS (FE-SRS) with MLHCF could be used for sensitive detection of dilute solutions.

Our proof-of-principle work demonstrated the feasibility of FE-SRS coupled with MLHCF. Although the signal enhancement compared with single-lens geometry was significant, the improvement in the detectability relative to the microscopy geometry was only moderate at the current stage [24]. The enhancement factor may be further improved in a few ways. Firstly, using a smaller fiber diameter (*d*) should increase the laser power density, and the overall SRS signal is estimated to be proportional to *d*^−2^. However, the XPM background may also be increased with a smaller diameter. Hence, the optimum diameter size will need to be experimentally decided to balance the SRS enhancement and XPM. Secondly, the optimum fiber length and enhancement factor is closely related to the absorption loss of the laser beams in liquids. Our simulation results showed that the optimum fiber length of SRS intensity was strongly dependent on the loss coefficient (α_p_), while it was less sensitive to the Raman gain coefficient (g_R_) (Figure S5). Since water and ordinary organic solvents have much less absorption in the visible wavelength range than in the NIR, FE-SRS is expected to be improved using visible light, which has additional gain in Raman scattering efficiency with shorter wavelengths. Therefore, systematic optimization of FE-SRS effect should consider the aforementioned factors, which requires the engineering of MLHCF and frequency-doubled SRS for further investigations. In addition, the small volume in the fiber (~6.7 µL) was advantageous for the measurement of valuable samples, but the current chamber in the home-made fiber adapter was spared quite a large volume (~192 µL), which could be reduced with improved design of the adapter.

In terms of applications, FE-SRS may open up new opportunities for sensing dilute solutions of biomolecules, drugs, etc. It will also be useful to rigorously compare the detectability of fiber-enhanced SRS with spontaneous Raman scattering. Especially for molecules with a low laser damage threshold, the much lower power density in the fiber waveguide may become a key advantage for stable measurements, compared with the common confocal Raman geometry where the focused laser spot is prone to cause sample degradation. Furthermore, CARS spectroscopy may be worth exploring with MLHCF, which is superior in a broader spectral range and improved spectroscopy with the help of advanced spectral analysis techniques [47].

## 4. Conclusions

In summary, we demonstrated the FE-SRS effect in liquid-filled MLHCF and showed its capability in quantitative detection of low-concentration analytes. The physical behavior and enhancement mechanism could be understood with our theoretical model. On the basis of the performance of our proposed configuration, we foresee the method having potential applications in rapid and sensitive Raman-based biosensing.

## Data Availability

The data that support the findings of this study are available from the corresponding author upon reasonable request.

## Supplementary Materials

The following supporting information can be downloaded at: https://www.mdpi.com/article/10.3390/bios12040243/s1. Supplementary Note S1: Theoretical modeling; Figure S1: Attenuation properties of the laser beams in MLHCF; Figure S2: Spontaneous Raman spectra of ethanol and DMSO; Figure S3: Theoretical relationship between SRS signal intensity and fiber length; Figure S4: The linear relationship between SRS intensity and analyte concentration; Figure S5: The relationship between the fiber length of the maximum SRS intensity and Raman gain coefficient and attenuation coefficient.

## References

[1] Raman C.V., Krishnan K.S. (1928). A new type of secondary radiation. Nature.

[2] Nie S.M., Emery S.R. (1997). Probing single molecules and single nanoparticles by surface-enhanced Raman scattering. Science.

[3] Frosch T., Yan D., Popp J. (2013). Ultrasensitive Fiber Enhanced UV Resonance Raman Sensing of Drugs. Anal. Chem..

[4] Zumbusch A., Holtom G.R., Xie X.S. (1999). Three-dimensional vibrational imaging by coherent anti-Stokes Raman scattering. Phys. Rev. Lett..

[5] Freudiger C.W., Min W., Saar B.G., Lu S., Holtom G.R., He C.W., Tsai J.C., Kang J.X., Xie X.S. (2008). Label-Free Biomedical Imaging with High Sensitivity by Stimulated Raman Scattering Microscopy. Science.

[6] Cheng J.X., Xie X.S. (2015). Vibrational spectroscopic imaging of living systems: An emerging platform for biology and medicine. Science.

[7] Evans C.L., Xu X.Y., Kesari S., Xie X.S., Wong S.T.C., Young G.S. (2007). Chemically-selective imaging of brain structures with CARS microscopy. Opt. Express.

[8] Cheng J.-X., Xie X.S. (2013). Coherent Raman Scattering Microscopy.

[9] Yan S., Cui S.S., Ke K., Zhao B.X., Liu X.L., Yue S.H., Wang P. (2018). Hyperspectral Stimulated Raman Scattering Microscopy Unravels Aberrant Accumulation of Saturated Fat in Human Liver Cancer. Anal. Chem..

[10] Fu D., Lu F.K., Zhang X., Freudiger C., Pernik D.R., Holtom G., Xie X.S. (2012). Quantitative Chemical Imaging with Multiplex Stimulated Raman Scattering Microscopy. J. Am. Chem. Soc..

[11] Zhang L., Shen S., Liu Z., Ji M. (2017). Label-Free, Quantitative Imaging of MoS2-Nanosheets in Live Cells with Simultaneous Stimulated Raman Scattering and Transient Absorption Microscopy. Adv. Biosyst..

[12] Yang Y., Yang Y., Liu Z., Guo L., Li S., Sun X., Shao Z., Ji M. (2021). Microcalcification-Based Tumor Malignancy Evaluation in Fresh Breast Biopsies with Hyperspectral Stimulated Raman Scattering. Anal. Chem..

[13] Zhang B.H., Xu H.L., Chen J., Zhu X.X., Xue Y., Yang Y.F., Ao J.P., Hua Y.H., Ji M.B. (2021). Highly specific and label-free histological identification of microcrystals in fresh human gout tissues with stimulated Raman scattering. Theranostics.

[14] Wei L., Chen Z.X., Shi L.X., Long R., Anzalone A.V., Zhang L.Y., Hu F.H., Yuste R., Cornish V.W., Min W. (2017). Super-multiplex vibrational imaging. Nature.

[15] Wei M., Shi L.Y., Shen Y.H., Zhao Z.L., Guzman A., Kaufman L.J., Wei L., Min W. (2019). Volumetric chemical imaging by clearing-enhanced stimulated Raman scattering microscopy. Proc. Natl. Acad. Sci. USA.

[16] Ji M., Orringer D.A., Freudiger C.W., Ramkissoon S., Liu X., Lau D., Golby A.J., Norton I., Hayashi M., Agar N.Y. (2013). Rapid, label-free detection of brain tumors with stimulated Raman scattering microscopy. Sci. Transl. Med..

[17] Ji M.B., Arbel M., Zhang L.L., Freudiger C.W., Hou S.S., Lin D.D., Yang X.J., Bacskai B.J., Xie X.S. (2018). Label-free imaging of amyloid plaques in Alzheimer’s disease with stimulated Raman scattering microscopy. Sci. Adv..

[18] Ao J.P., Feng Y.Q., Wu S.M., Wang T., Ling J.W., Zhang L.W., Ji M.B. (2020). Rapid, 3D Chemical Profiling of Individual Atmospheric Aerosols with Stimulated Raman Scattering Microscopy. Small Methods.

[19] Fu D., Zhou J., Zhu W.S., Manley P.W., Wang Y.K., Hood T., Wylie A., Xie X.S. (2014). Imaging the intracellular distribution of tyrosine kinase inhibitors in living cells with quantitative hyperspectral stimulated Raman scattering. Nat. Chem..

[20] Hu F., Shi L., Min W. (2019). Biological imaging of chemical bonds by stimulated Raman scattering microscopy. Nat. Methods.

[21] Ao J., Fang X., Miao X., Ling J., Kang H., Park S., Wu C., Ji M. (2021). Switchable stimulated Raman scattering microscopy with photochromic vibrational probes. Nat. Commun..

[22] Hollon T.C., Pandian B., Adapa A.R., Urias E., Save A.V., Khalsa S.S.S., Eichberg D.G., D’Amico R.S., Farooq Z.U., Lewis S. (2020). Near real-time intraoperative brain tumor diagnosis using stimulated Raman histology and deep neural networks. Nat. Med..

[23] Zhang L., Zou X., Huang J., Fan J., Sun X., Zhang B., Zheng B., Guo C., Fu D., Yao L. (2021). Label-Free Histology and Evaluation of Human Pancreatic Cancer with Coherent Nonlinear Optical Microscopy. Anal. Chem..

[24] He R.Y., Liu Z.P., Xu Y.K., Huang W., Ma H., Ji M.B. (2017). Stimulated Raman scattering microscopy and spectroscopy with a rapid scanning optical delay line. Opt. Lett..

[25] Levenson M.D. (1982). Introduction to Nonlinear Laser Spectroscopy.

[26] Boyd R.W. (2008). Nonlinear Optics.

[27] Hanf S., Keiner R., Yan D., Popp J., Frosch T. (2014). Fiber-Enhanced Raman Multigas Spectroscopy: A Versatile Tool for Environmental Gas Sensing and Breath Analysis. Anal. Chem..

[28] Yan D., Popp J., Frosch T. (2017). Analysis of Fiber-Enhanced Raman Gas Sensing Based on Raman Chemical Imaging. Anal. Chem..

[29] Jin Z., Chu Q., Xu W., Cai H., Ji W., Wang G., Lin B., Zhang X. (2018). All-Fiber Raman Biosensor by Combining Reflection and Transmission Mode. IEEE Photonics Technol. Lett..

[30] Cai H., Yu X., Chu Q., Jin Z., Lin B., Wang G. (2019). Hollow-core fiber-based Raman probe extension kit for in situ and sensitive ultramicro-analysis. Chin. Opt. Lett..

[31] Chu Q., Jin Z., Yu X., Li C., Zhang W., Ji W., Lin B., Shum P.P., Zhang X., Wang G. (2019). Volumetric enhancement of Raman scattering for fast detection based on a silver-lined hollow-core fiber. Opt. Express.

[32] Knebl A., Yan D., Popp J., Frosch T. (2018). Fiber enhanced Raman gas spectroscopy. TrAC Trends Anal. Chem..

[33] Yiou S., Delaye P., Rouvie A., Chinaud J., Frey R., Roosen G., Viale P., Fevrier S., Roy P., Auguste J.L. (2005). Stimulated Raman scattering in an ethanol core microstructured optical fiber. Opt. Express.

[34] Benabid F., Knight J.C., Antonopoulos G., Russell P.S.J. (2002). Stimulated Raman scattering in hydrogen-filled hollow-core photonic crystal fiber. Science.

[35] Benabid F., Couny F., Knight J.C., Birks T.A., Russell P.S. (2005). Compact, stable and efficient all-fibre gas cells using hollow-core photonic crystal fibres. Nature.

[36] Eravuchira P.J., Banchelli M., D’Andrea C., de Angelis M., Matteini P., Gannot I. (2020). Hollow core photonic crystal fiber-assisted Raman spectroscopy as a tool for the detection of Alzheimer’s disease biomarkers. J. Biomed. Opt..

[37] Yan D., Popp J., Pletz M.W., Frosch T. (2017). Highly Sensitive Broadband Raman Sensing of Antibiotics in Step-Index Hollow-Core Photonic Crystal Fibers. Acs Photonics.

[38] Wolf S., Frosch T., Popp J., Pletz M.W., Frosch T. (2019). Highly Sensitive Detection of the Antibiotic Ciprofloxacin by Means of Fiber Enhanced Raman Spectroscopy. Molecules.

[39] Yan D., Frosch T., Kobelke J., Bierlich J., Popp J., Pletz M.W., Frosch T. (2018). Fiber-Enhanced Raman Sensing of Cefuroxime in Human Urine. Anal. Chem..

[40] Antonopoulos G., Benabid F., Birks T.A., Bird D.M., Knight J.C., Russell P.S.J. (2006). Experimental demonstration of the frequency shift of bandgaps in photonic crystal fibers due to refractive index scaling. Opt. Express.

[41] James T.M., Rupp S., Telle H.H. (2015). Trace gas and dynamic process monitoring by Raman spectroscopy in metal-coated hollow glass fibres. Anal. Methods.

[42] Andreana M., Le T., Drexler W., Unterhuber A. (2019). Ultrashort pulse Kagome hollow-core photonic crystal fiber delivery for nonlinear optical imaging. Opt. Lett..

[43] He R., Xu Y., Zhang L., Ma S., Wang X., Ye D., Ji M. (2017). Dual-phase stimulated Raman scattering microscopy for real-time two-color imaging. Optica.

[44] Smarandache A., Moreno-Moraga J., Staicu A., Trelles M., Pascu M.L. (2012). Applications of Polidocanol in Varicose Vein Treatment Assisted by Exposure to Nd: YAG Laser Radiation. Nd YAG Laser.

[45] Emin A., Hushur A., Mamtimin T. (2020). Raman study of mixed solutions of methanol and ethanol. AIP Adv..

[46] Agrawal G.P. (2001). Nonlinear Fiber Optics. Lect. Notes Phys..

[47] Camp C.H., Lee Y.J., Heddleston J.M., Hartshorn C.M., Walker A.R.H., Rich J.N., Lathia J.D., Cicerone M.T. (2014). High-speed coherent Raman fingerprint imaging of biological tissues. Nat. Photonics.

