# Supplementary material for: Fiber-Enhanced Stimulated Raman Scattering and Sensitive Detection of Dilute Solutions
Source: Biosensors (Basel). 2022 Apr 14;12(4):243. doi: 10.3390/bios12040243 (PMC9028131; doi:10.3390/bios12040243)
Supplement: Supplementary file 1 [file biosensors-12-00243-s001.zip › biosensors-1667937-supplementary.pdf]

Supplementary Materials

Fiber-enhanced stimulated Raman scattering and sensitive detection of dilute solutions

Li Guo[1], Jing Huang[1], Yaxin Chen[1], Bohan Zhang[1], Minbiao Ji[1,2]*

[1] State Key Laboratory of Surface Physics and Department of Physics, Human Phenome Institute, Academy for Engineering and Technology, Key Laboratory of Micro and Nano Photonic Structures (Ministry of Education), Fudan University, Shanghai 200433, China

[2] Yiwu Research Institute of Fudan University, Chengbei Road, Yiwu City 322000, China

***CORRESPONDENCE:** minbiaoj@fudan.edu.cn

Supplementary Materials Note S1. Theoretical modeling

If the pump beam was launched at the fiber input with the Stokes beam, both the fiber losses and the nonlinear interaction between the pump and Stokes beams need to be taken into account. The SRS process was decided by the following set of two coupled equations:

$$\frac{\mathrm{d}I_\mathrm{p}}{\mathrm{dz}} = -\frac{\omega_\mathrm{p}}{\omega_\mathrm{s}} \mathrm{g}_R I_\mathrm{p} I_\mathrm{s} - \alpha_\mathrm{p} I_\mathrm{p} \tag{1}$$

$$\frac{\mathrm{d}I_\mathrm{s}}{\mathrm{dz}} = \mathrm{g}_R I_\mathrm{p} I_\mathrm{s} - \alpha_\mathrm{s} I_\mathrm{s} \tag{2}$$

where g_R is the Raman-gain coefficient.

An approximate expression for I_s and I_p can be acquired by solving Eqs(1) and (2) analytically with the assumption $\alpha_p = \alpha_s \equiv \alpha$ and the results are

$$I_p(z) = I_p(0)(1+\rho\frac{I_s(0)}{I_p(0)})e^{-\alpha z} \Big/ G(z) \tag{3}$$

$$I_s(z) = I_s(0)(1+\rho\frac{I_s(0)}{I_p(0)})\exp[F(z)-\alpha z] \Big/ G(z) \tag{4}$$

where

$$F(z) = \rho g_R(\frac{I_p(0)}{\rho} + I_s(0))\left(1-e^{-\alpha z}\right) \Big/ \alpha$$

$$G(z) = 1+\rho\frac{I_s(0)}{I_p(0)}e^{-F(z)}$$

where $I_s(0)$ and $I_p(0)$ are the initial conditions describing the intensities of Stokes and pump beams at $z=0$ respectively.

If pump beam is launched at the input end without the Stokes beam, then only the power losses need to be considered. The decrease of the pump beam is governed by

$$\frac{\mathrm{d}I_{\mathrm{p}}^{'}}{\mathrm{dz}} = -\alpha I_{\mathrm{p}}^{'} \tag{5}$$

This equation is easy to solve, and the result can be written by using the initial conditions $I_p(0)$ as

$$I_{\mathrm{p}}^{'}(z) = I_p(0)e^{-\alpha z} \tag{6}$$

Thus, SRL signal can be described by

$$I_{SRL} = \Delta I(\omega_p) = I_{\mathrm{p}}^{'} - I_{\mathrm{p}} = I_p(0)e^{-\alpha z} - I_p(0)(1+\rho\frac{I_s(0)}{I_p(0)})e^{-\alpha z} \Big/ G(z) \tag{7}$$

And the fractional change is:

$$I_{SRL} / I_{\mathrm{p}}^{'} = 1-(1+\rho\frac{I_s(0)}{I_p(0)})/G(z) \tag{8}$$

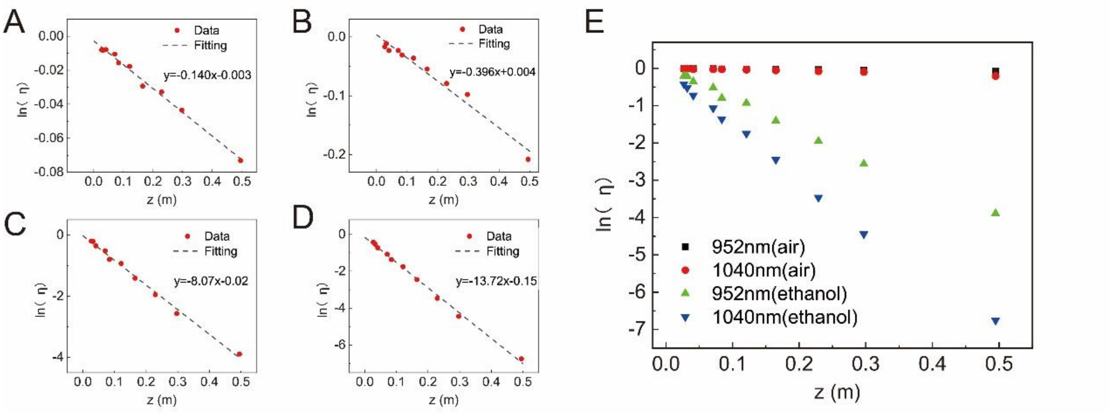

Figure S1. Attenuation of pump and Stokes beams in MLHCF. (A) ~ (B) 952nm pump and 1040nm Stokes beams in air core of the empty MLHCF. (C) ~ (D) 952nm pump and 1040nm Stokes beams in liquid core of the ethanol-filled MLHCF. (E) Comparison diagram of 952nm pump and 1040nm Stokes beams in empty and ethanol-filled MLHCF.

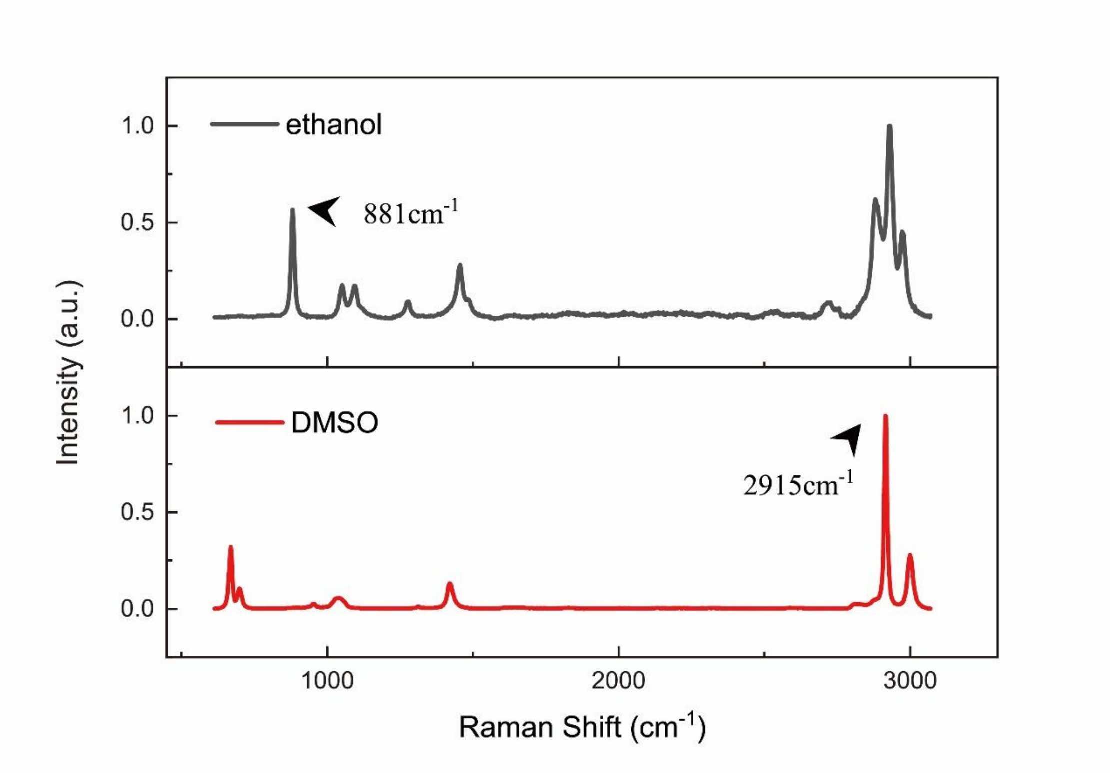

Figure S2. Spontaneous Raman spectra of ethanol and DMSO.

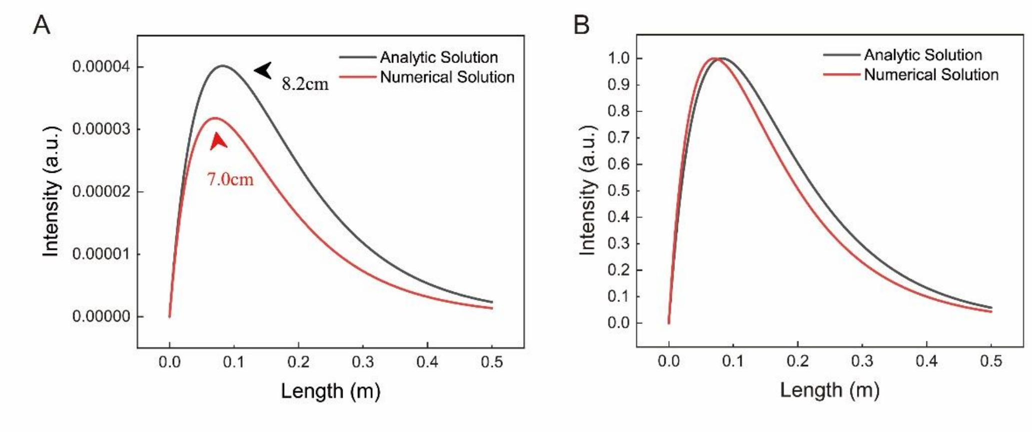

Figure S3. Theoretical relationship between SRS signal intensity and fiber length. (A)Analytic solution (black line) and numerical solution (red line). (B) The normalized analytic solution (black line) and numerical solution (red line).

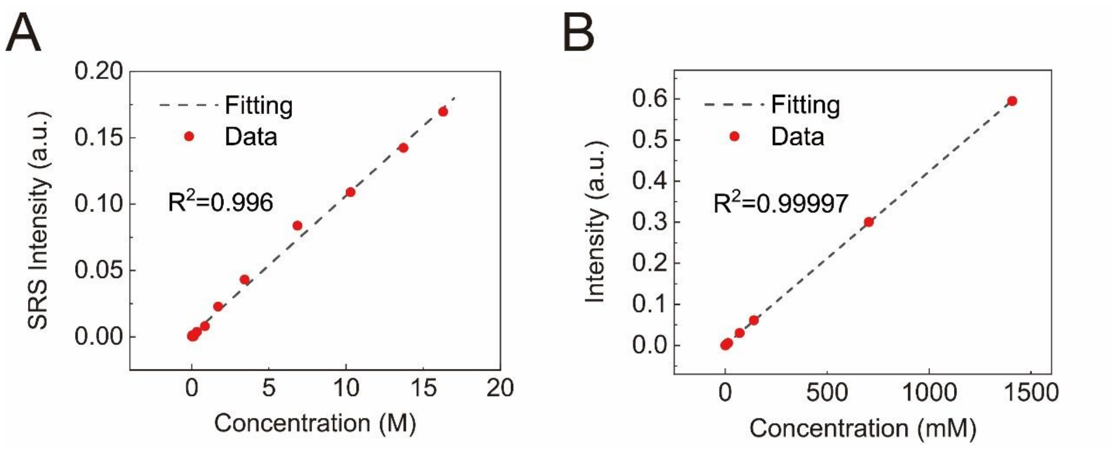

Figure S4. The linear relationship between SRS intensity and analyte concentrations in MLHCF. (A) Ethanol (881 cm^{-1}); (B) DMSO (2915 cm^{-1}) .

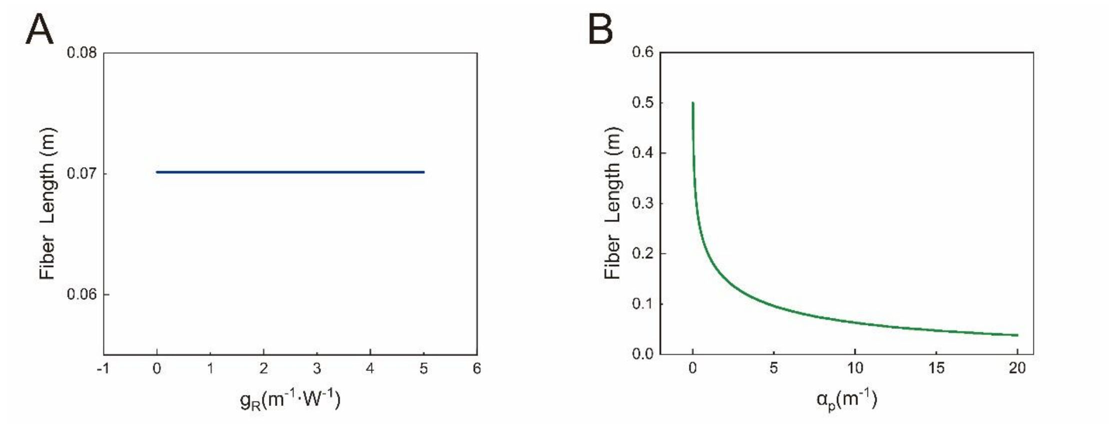

Figure S5. The relationship between the fiber length of the maximum SRS intensity and (A) the Raman gain coefficient g_R and (B) the pump beam attenuation coefficient α_p.
